# Annual Reproductive Cycle and Unusual Embryogenesis of a Temperate Coral in the Mediterranean Sea

**DOI:** 10.1371/journal.pone.0141162

**Published:** 2015-10-29

**Authors:** Chiara Marchini, Valentina Airi, Roberto Fontana, Giada Tortorelli, Marta Rocchi, Giuseppe Falini, Oren Levy, Zvy Dubinsky, Stefano Goffredo

**Affiliations:** 1 Marine Science Group, Department of Biological, Geological and Environmental Sciences, University of Bologna, Via F. Selmi 3, 40126 Bologna, Italy; 2 Department of Chemistry “G. Ciamician”, University of Bologna, Via F. Selmi 2, 40126 Bologna, Italy; 3 The Mina and Everard Goodman Faculty of Life Sciences, Bar-Ilan University, Ramat Gan 52900, Israel; Biodiversity Research Center, Academia Sinica, TAIWAN

## Abstract

The variety of reproductive processes and modes among coral species reflects their extraordinary regeneration ability. Scleractinians are an established example of clonal animals that can exhibit a mixed strategy of sexual and asexual reproduction to maintain their populations. This study provides the first description of the annual reproductive cycle and embryogenesis of the temperate species *Caryophyllia inornata*. Cytometric analyses were used to define the annual development of germ cells and embryogenesis. The species was gonochoric with three times more male polyps than female. Polyps were sexually mature from 6 to 8 mm length. Not only females, but also sexually inactive individuals (without germ cells) and males were found to brood their embryos. Spermaries required 12 months to reach maturity, while oogenesis seemed to occur more rapidly (5–6 months). Female polyps were found only during spring and summer. Furthermore, the rate of gamete development in both females and males increased significantly from March to May and fertilization was estimated to occur from April to July, when mature germ cells disappeared. Gametogenesis showed a strong seasonal influence, while embryos were found throughout the year in males and in sexually inactive individuals without a defined trend. This unusual embryogenesis suggests the possibility of agamic reproduction, which combined with sexual reproduction results in high fertility. This mechanism is uncommon and only four other scleractinians (*Pocillopora damicornis*, *Tubastraea diaphana*, *T*. *coccinea* and *Oulastrea crispata*) have been shown to generate their broods asexually. The precise nature of this process is still unknown.

## Introduction

Reproductive biology is a key feature of an organism’s life strategy [1] and is fundamental to understand the population structure and dynamics of sessile animals [2], which are an important component of aquatic communities. Corals are modular organisms that can potentially lead to a variety of reproductive processes and modes, reflecting their extraordinary regeneration ability, developmental plasticity, and adaptability [3,4]. However, for reproduction, there are essentially only four combinations of reproductive patterns: propagation mode (sexual or asexual), sexuality (hermaphroditic or gonochoric), reproductive mode (broadcasting or brooding), and embryonic development (coeloblastula or stereoblastula) [4]. These organisms can display a mixed propagation mode of sexual and asexual reproduction in order to preserve their populations [5]. Simultaneous mixed reproduction is rare in animals and is often described as the “best-of-both-worlds” scenario that can help organisms adapt to changing environments [6]. Sexual reproduction requires the production of gametes, fertilization, embryo development, a larval phase and enables genetic recombination and production of new genotypes. This genotypically different lineage might enable a wide dispersion or recolonization of more heterogeneous habitats, increasing the fitness and survival of the species [7,8]. Asexual reproduction may take place via colony fragmentation, colony fission, longitudinal and transverse division, polyp expulsion or polyp “bail-out”, budding and, in rare cases, the production of brooded embryos spreading successful genotypes without mating [4]. This clonal line might contribute to keeping populations inside the area of the parental habitat, thus propagating well-adapted genotypes at the local level [8]. It may be also an adaptation that allows the exploitation of newly available substrata after a disturbance event [9].

Concerning sexuality, most of the scleractinians are hermaphrodites and only 26% of the studied species are described as gonochoric [3,4]. The hermaphroditism normally is simultaneous, but there are some forms of hermaphroditism more complex to detect as the cyclic sequential in the same breeding season (as has been described for three deep species of the genus *Caryophyllia*) [10] and the protandrous or protogynous sequential during the life. *Lobactis scutaria* and *Lithophyllon repanda* are predominantly male at small sizes whereas large individuals are all females, suggesting that these fungiids are protandrous hermaphrodites [11,12]. Additionally, *Ctenactis echinata* is a protandrous species but has the capacity for bidirectional sex change between the years as occurs in dioecious plants that display a labile sexuality in response to energetic and/or environmental constraints [12].

Fertilization can be either internal when the embryo is formed and develops within the polyp and is released as a motile planula (brooding), or external when the embryo develops in the water column (broadcast spawning); the first condition is less common within the order Scleractinia and represents the 16% of the total number of known coral species [3,4]. Very few brooders can produce planulae by asexual processes, indeed, it has been shown only in some populations of *Pocillopora damicornis* [13], sometimes in combination with gametogenetic activity [9,14,15], in *Tubastraea diaphana* [16], *T*. *coccinea* [16,17], and *Oulastrea crispata* [18,19]. These scleractinians were also found to be pioneer species, colonizing unpredictable, short-lived or unexploited habitats as oil and gas platforms [19,20].

The reproductive cycle can be regulated by several environmental factors such as seawater temperature, photoperiod, wind or current patterns, lunar cycles of night irradiance, food availability and seasonal rainfall [1,4]. In particular, photoperiod (therefore solar radiation) and seawater temperature are not mutually exclusive events. In fact, in the Mediterranean Sea there are marked seasonal patterns of seawater temperature driven by photoperiod and irradiance cycles distinctive of temperate latitudes [1]. However, while several studies have shown that seawater temperature strongly influences gametogenesis [17,21–25], the potential role of photoperiod has so far been overlooked.

Although reproduction of scleractinians has been thoroughly studied in the last decades [12,21,22,26–34], the great variety of reproductive strategies within this group is not yet entirely known and even less is known about asexual patterns. Furthermore, knowledge on the reproductive biology of Mediterranean scleractinian corals is scarce and exclusively linked to aspects of the sexual propagation of *Balanophyllia europaea* [35–39], *Leptopsammia pruvoti* [2,40], *Cladocora caespitosa* [41,42] and *Astroides calycularis* [43–45].

This manuscript describes, for the first time, the quantitative aspects (sex ratio, size of individuals at sexual maturity, fecundity, and seasonal patterns of gonadal development and fertility) of the annual reproductive cycle in the Mediterranean solitary coral *Caryophyllia inornata* (S1 Fig; Duncan, 1878) at Elba Isle (Italy). Some aspects of the reproductive biology of this species have already been described, revealing a gonochoric sexuality and a brooding reproductive mode, driven by an unusual pattern of embryogenesis in which embryos are found in females, males and sexually inactive individuals throughout the year, suggesting a possible asexual origin of the embryos [46].

## Materials and Methods

### Ethic Statement

According to the European normative (2010/63/EU of 8 August 2010) on the protection of animals used for scientific purposes, there is no active conservation measure for the Mediterranean coral *Caryophyllia inornata*. The species is not protected in Italy, nor it is subject to any regulations. Hence, no permit was needed to collect samples. For this study, sampling was limited strictly to the number necessary and performed where the species is characterized by a high population density to minimize the impact of removing individuals and preserve both the demographic and genetic structure of the natural populations.

### Study species, sample collection and environmental parameters

The solitary coral *Caryophyllia inornata* is distributed in the Mediterranean Sea [47] and extends up to the Northeastern Atlantic coasts [48], from the Canary Islands to the Southern coast of the United Kingdom [47]. It colonizes caves, walls and wrecks, from the surface down to 100 m depth in dimly lit or dark environments, representing one of the main species that populate the walls and the vaults of caves and in some cases is the dominant species [49].

Polyps were collected from an aircraft wreck at Elba Isle (42°45’N, 10°24’E), during 18 monthly samplings from May 2009 to October 2010. A minimum of 15 polyps were collected randomly each month at a depth of 12–15 m by SCUBA diving. The population density in the sampling site was 6025 ± 898 (mean ± SE) individuals m^-2^ with a percentage cover of 15.3 ± 2.5% (mean ± SE) [50].

Photoperiod data were obtained from an online database (http://www.eurometeo.com). Water temperature (°C) was continuously recorded every three hours by digital sensors (I-Button DS1921H, Maxim Integrated Products) placed at the depth and site of collection for the entire sampling period. A linear regression was produced between DT (Depth Temperature; °C) and SST (Sea Surface Temperature; °C) data to estimate temperatures during periods in which sensors were lost due to bad weather conditions. In this study we considered the monthly average DT of almost two years of sampling (n = 18 monthly temperatures).

Polyps were fixed in saturated formalin solution (10% formaldehyde and 90% seawater; the solution was saturated with calcium carbonate) and transferred to the laboratories for histological analysis.

### Biometric and histological analysis

Biometric analyses were performed on 158 polyps by measuring length (L, maximum axis of the oral disc), width (l, minimum axis of the oral disc) and height (h, oral–aboral axis) of each sampled polyp. The volume (V) of the individual polyp was calculated using the formula V = h * (L/2) * (l/2) * π [37].

Polyps were post-fixed in Bouin solution. After decalcification in EDTA and dehydration in a graded alcohol series from 80% to 100%, polyps were embedded in paraffin and serial transverse sections were cut at 7 μm intervals along the oral-aboral axis, from the oral to the aboral poles. Tissues were then stained with Mayer’s haematoxylin and eosin [37].

### Cytohistometric analysis

Cytohistometric observations were performed with an optical microscope using the software NIKON NIS-Elements D 3.2. The maximum and minimum diameters of the oocytes in nucleated sections and spermaries were measured and classified into developmental stages according to earlier studies on gametogenesis in scleractinians [11,37,51–54]. The presence of embryos in the gastrovascular cavity and mesenterial septa was recorded, and their stage of maturation identified [2,35]. The size of each reproductive element was determined as the mean of the two diameters [2,43].

### Definitions

In accordance with the sexuality described by Goffredo *et al* [46], based on the type of germ cells observed and the presence or absence of embryos, 5 reproductive states have been identified: sexually active individuals that present gametogenetic activity (i.e., females with embryos, males, and males with embryos) and sexually inactive individuals, without germ cells (i.e., inactive individuals and inactive individuals with embryos).

The following reproductive parameters were determined: a) *size at sexual maturity*, defined as the length at which 50% of the analyzed polyps developed spermaries or oocytes; b) *fecundity*, defined as the number of mature oocytes produced per body volume unit (100 mm^3^) per reproductive season; c) *gonadal index*, defined as the percentage of body volume occupied by germ cells [37]; d) *fertility*, defined as the number of embryos per body volume unit (100 mm^3^).

## Results

### Sexuality and reproductive mode

The analysis of 158 polyps confirmed that *Caryophyllia inornata* is gonochoric and brooder [46]. The sex ratio of sexually active polyps was significantly different from 1 with a 1:3.5 male biased ratio (chi-square test, χ^2^ = 20.43, df = 1, p < 0.001).

**Fig 1:**
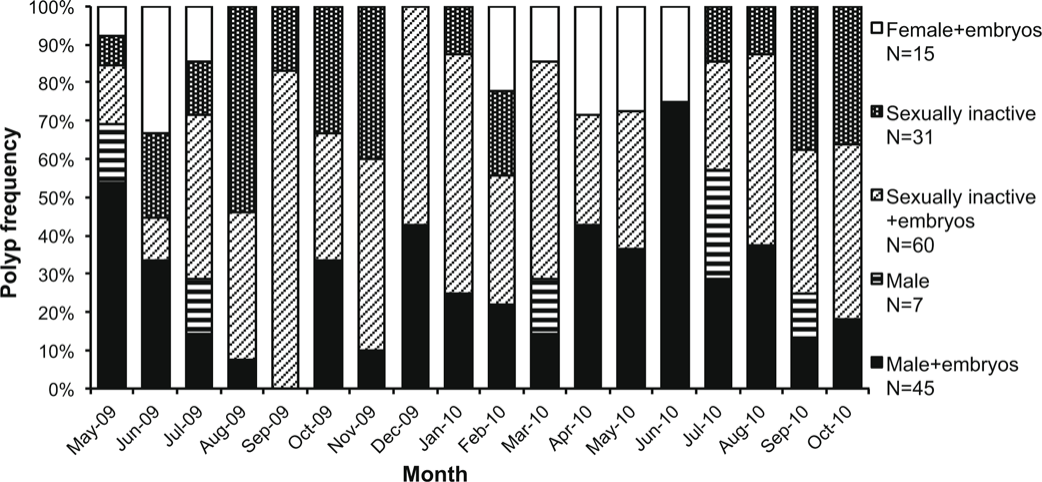
Monthly frequency of the 5 reproductive states. Monthly frequency of the 5 reproductive states (female with embryos, inactive individual, inactive individual with embryos, male and male with embryos) characterizing the population of Elba Isle, between May 2009 and October 2010 (N = 158).

Embryos were found in all monthly samples and inside females, males, and inactive individuals (Fig 1) [46]. All 15 females had embryos (L = 7.9 ± 0.4 mm; V = 366 ± 47 mm^3^; mean ± SE). Of the 52 males, 45 had embryos (L = 8.2 ± 0.3 mm; V = 363 ± 31 mm^3^; mean ± SE) and 7 were without embryos (L = 6.5 ± 0.4 mm; V = 219 ± 27 mm^3^; mean ± SE). Of the 91 inactive polyps, 60 had embryos (L = 7.9 ± 0.3 mm; V = 341 ± 29 mm^3^; mean ± SE) and 31 did not show embryos (L = 5.5 ± 0.4 mm; V = 171 ± 40 mm^3^; mean ± SE).

**Fig 2:**
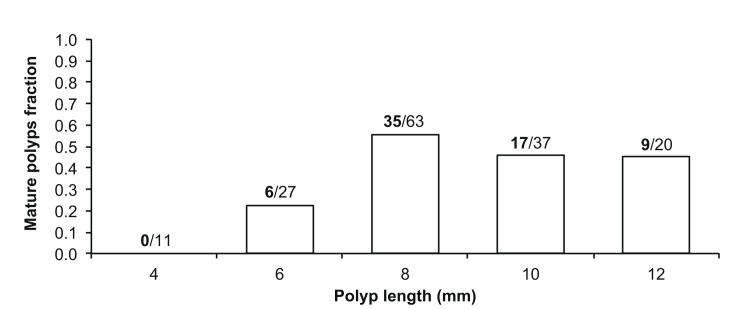
Fraction of sexually mature individuals per size class (mm). Fraction of sexually mature individuals per size class in millimeters, collected at Elba Isle. The values above the bars indicate the number of sexually mature polyps (bold) out of the number of polyps analyzed per size class (N = 158).

Polyps up to 6 mm in length were immature and size at sexual maturity ranged from 6 to 8 mm in length (Fig 2). According to biometric analyses a polyp in this category has l = 5–7 mm, h = 5–6 mm, V = 146–206 mm^3^. The frequency of sexually mature polyps decreased in larger size classes (Fig 2).

### Annual reproductive cycle

**Fig 3:**
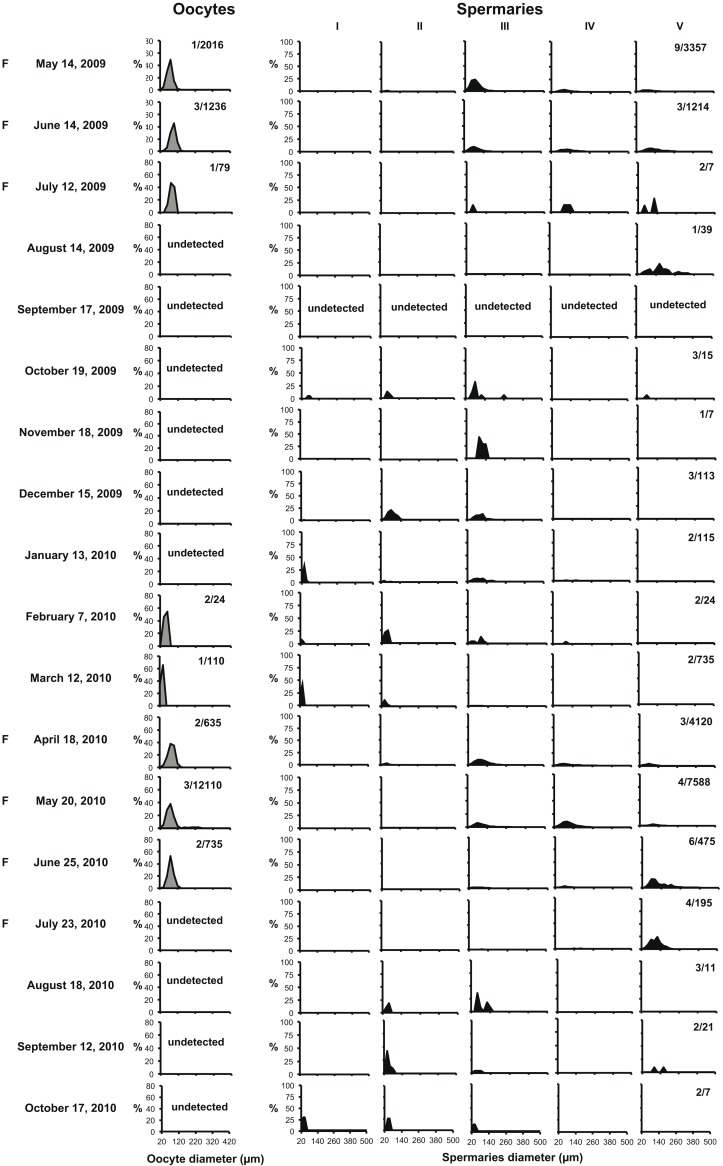
Oocytes and spermaries size-frequency distribution. Size-frequency distribution of oocytes and of the five stages of spermary maturation in monthly samples collected at Elba Isle from May 2009 to October 2010. Values reported indicate the number of polyps/the total number of oocytes or spermaries measured per monthly sample. F = fertilization period.

**Fig 4:**
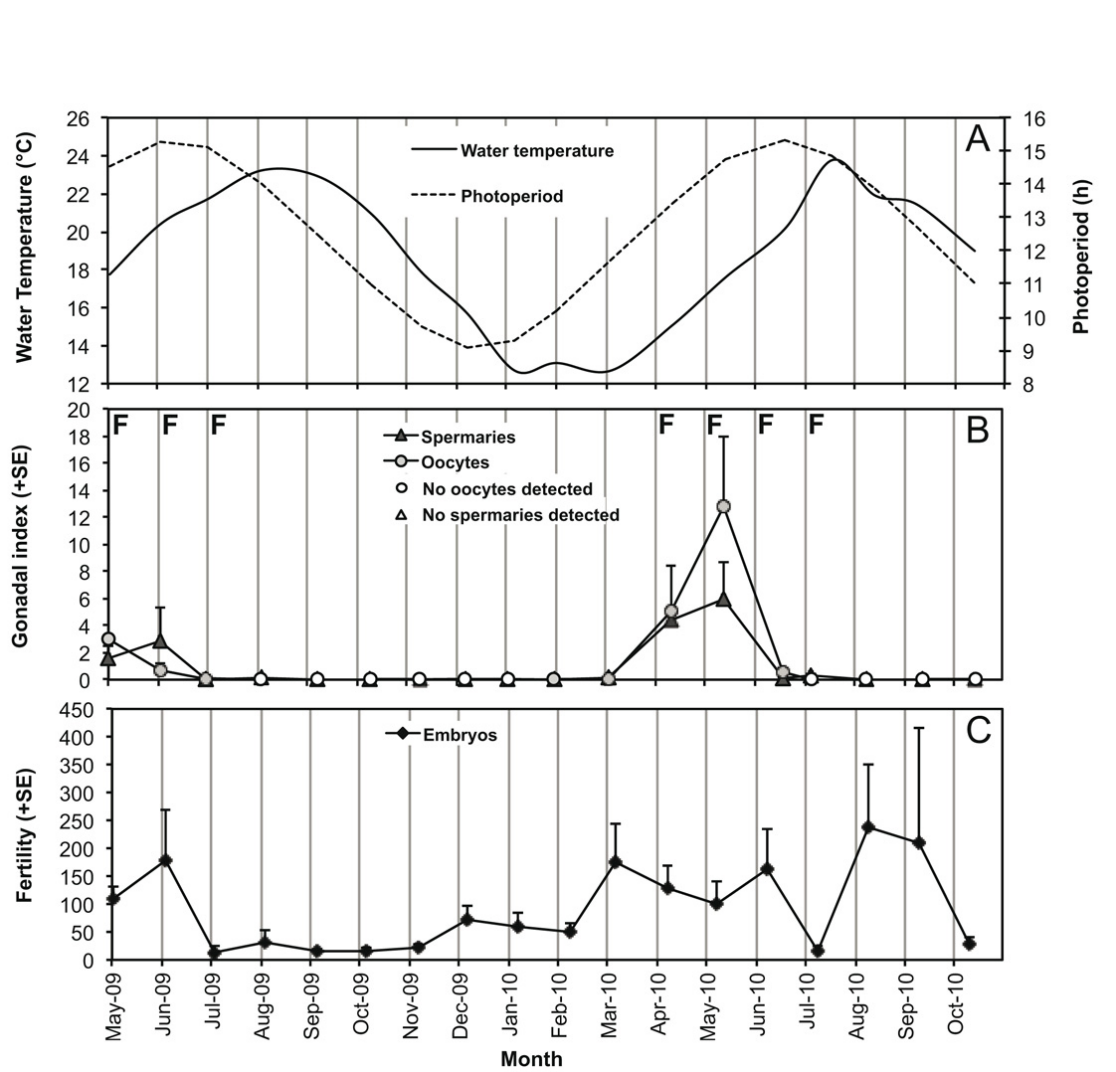
Variation in water temperature, photoperiod, gamete development and fertility. Variation in water temperature and photoperiod (A), gamete development (monthly mean + SE; B), and total fertility (monthly mean + SE; C) from May 2009 to October 2010 at Elba Isle. F = fertilization period.

Female polyps were observed between February and July, while males were found during the entire year (Figs 1 and 3). This suggests that the oogenesis process requires less time to reach the final stage of maturation than spermatogenesis, which needed about 12 months (Fig 3). Gonadal size of both females and males increased significantly from March until May, when both photoperiod and water temperature increased after the minimum of the year (Fig 4A and 4B). Fertilization took place from April to July, when photoperiod was the longest of the year (Fig 4A and 4B). Immediately after the fertilization period, we observed the emptying of spermaries and we did not register the presence of oocytes (Fig 3). During the autumn months following the fertilization period, we observed the development of early stages of spermaries maturation in males (Fig 3).

### Size of mature oocytes and fecundity

All the oocytes of *Caryophyllia inornata* reached maturity during the period from February to July, since we observed their disappearance after fertilization. Mature oocyte size was 69.7 μm (SE = 0.1) and ranged from 12 μm to 382 μm. We found a mean fecundity of 20’106 (SE = 11’715) mature oocytes in averaged-sized females of L = 7.9 mm (SE = 0.4), corresponding to l = 7.0 mm (SE = 0.4), h = 8.0 mm (SE = 0.4), V = 366 mm^3^ (SE = 47), N = 15 polyps collected during the period of gonadal development (Fig 4B).

### Fertility

**Fig 5:**
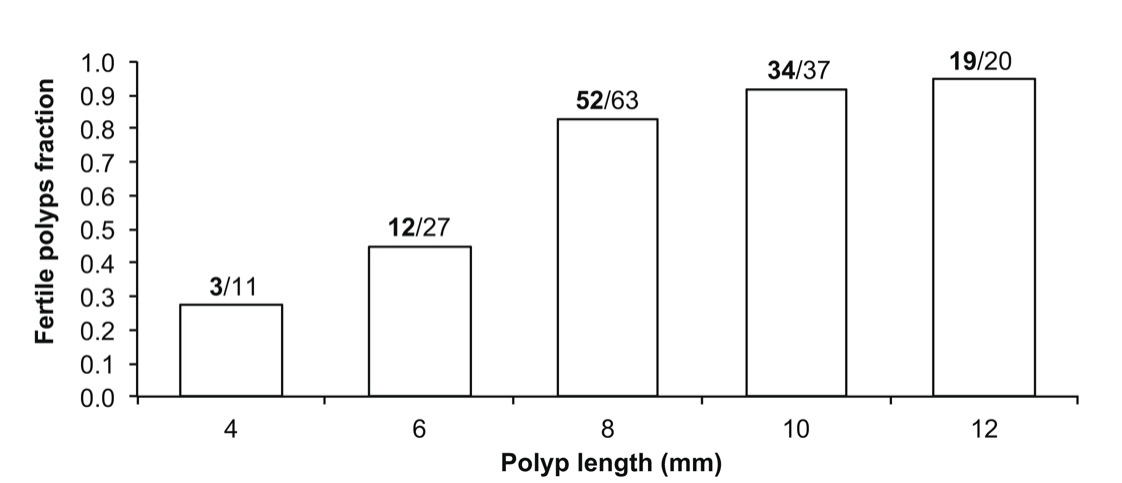
Fraction of fertile individuals per size class (mm). Fraction of fertile individuals per size class in millimeters, collected at Elba Isle. The values above the bars indicate the number of sexually mature polyps (bold) out of the number of polyps analyzed per size class (N = 158).

**Fig 6:**
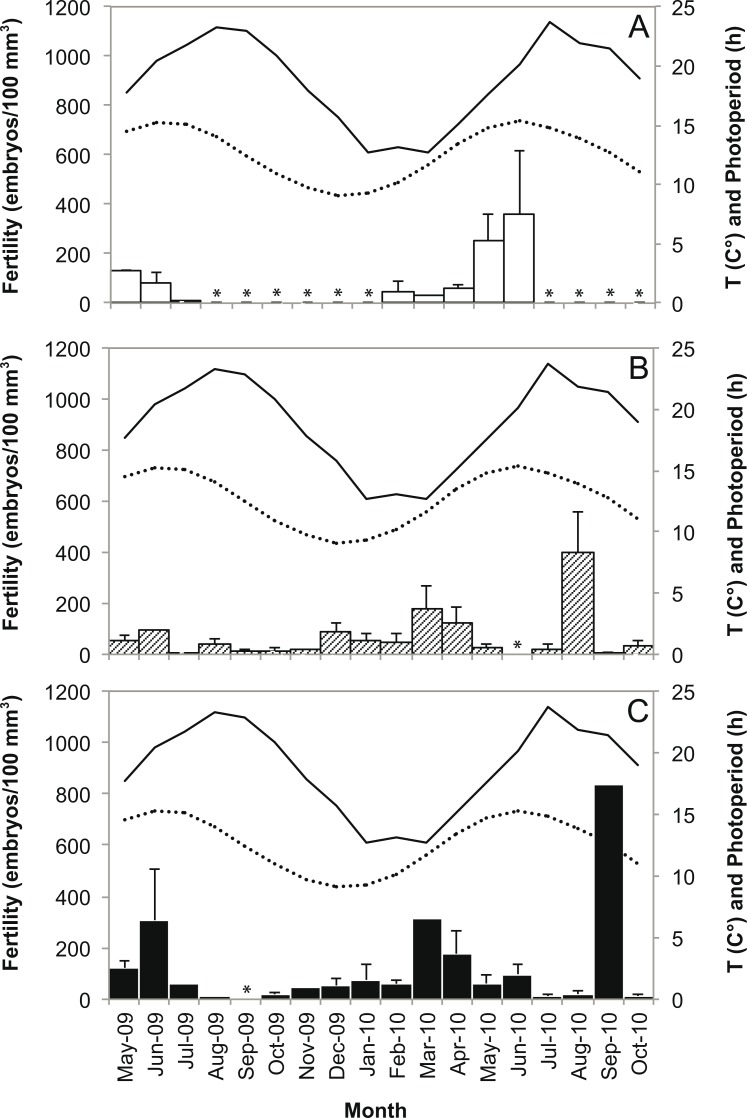
Diagram of the relationship between environmental parameters and fertility. Diagram of the relationship between water temperature (solid line), photoperiod (dotted line) and monthly mean fertility (bars) of females (A), sexually inactive individuals (B) and males (C) from May 2009 to October 2010. Error bars are standard errors (SE). * Reproductive state not detected.

Polyps up to 6 mm in length were not fertile and size at embryo production ranged from 6 to 8 mm in length (Fig 5). A continuous production of embryos in different stages of development (early embryos, intermediate and advanced stereogastrulae) [46] was observed during the entire year (Fig 4C). The fertility of females increased significantly from April to June, the same period in which gonadal development increased and fertilization occurred (Figs 4B and 4C and 6A). Embryos inside males and sexually inactive individuals were observed in all sampling months without a clear relation with seasonal variations of water temperature and photoperiod (Fig 6B and 6C).

## Discussion

### Sexuality and reproductive mode

This study provides the first description of the quantitative aspects of the annual reproductive cycle and embryogenesis of the temperate species *Caryophyllia inornata*.

*C*. *inornata* is gonochoric and brooder, as previously described for this species [46]. Histological analyses confirmed that no polyps showed simultaneous male and female gametes in different stages of development, excluding the possibility of a cyclical hermaphroditism, as reported for the three deep species of the genus *Caryophyllia* [10]. Also, protandrous or protogynous sequential hermaphroditism can be excluded, as the size of male and female individuals was not significantly different [46].

The male biased sex ratio observed in *C*. *inornata* could be explained by a clonal propagation where male clones are more likely to reproduce asexually than females, as has been reported in some solitary scleractinians of the Fungidae family: *Diaseris distorta*, *Lobactis scutaria*, *Lithophyllon concinna* and *Fungia fungites* [11,23,55]. A male biased sex ratio may also increase fertilization success, resulting in an advantage for sessile gonochoric corals with internal or surface fertilization [56–58]. Within the family Caryophylliidae, an agamic propagation by unequal intratentacular budding was observed in the colonial coral *Lophelia pertusa* [59–61]. This cold-water scleractinian also displays sexual reproduction, following an annual cycle of gametogenesis [62]. Evidence of an asexual production of brooded embryos in combination with gametogenetic activity, as it might occur for *C*. *inornata*, has been demonstrated in some populations of *Pocillopora damicornis*, in Western Australia, Hawaii, and southern Japan [6,9,15,16,63–67]. This strategy has been observed in other tropical scleractinians like *Tubastraea diaphana* [16], *T*. *coccinea* [17] and *Oulastrea crispata*, which can also produce asexual embryos during periods when gametogenesis is not occurring [18,19]. This mixed reproductive strategy might allow colonization of new structures in the sea, in a relatively short period of time [19]. The Australian sea anemone *Actinia tenebrosa* [68,69] and the tropical *A*. *bermudensis* [70] brood embryos genetically identical to the parent. The same pattern of embryogenesis was observed in the temperate *A*. *equina*, which displays asexual brooded embryos while undergoing a (regular) gametogenetic cycle and reveals genetic variation at isozyme loci, providing clear evidence that sexual reproduction also occurs [71–74]. However, to date, none of these species have been shown to use sexual reproduction to produce brooded larvae. Instead, sexual larvae could be generated by broadcast spawning and external fertilization [72,74–76], probably to produce widely dispersed planktonic progeny [7].

Reaching sexual maturity is a process which depends on size and age of the organism and is one of the main components of reproductive biology [77]. *C*. *inornata* reached sexual maturity between 6 and 8 mm in length. The fraction distribution of sexually mature individuals has a bell-like shape, where both smaller size and larger size individuals tended to not produce germ cells. Smaller polyps may be immature individuals without the ability to produce gametes, while larger polyps may be sexually old individuals that preserve the ability to produce agamic embryos. In fact, it is possible that this species, after reaching a certain size/age, is affected by senescence [46] leading to a progressive decline in metabolic functions and to an increase in the mortality rate [78]. This phenomenon was demonstrated for the colonial coral *Stylophora pistillata* which shows a significant decrease in the rate of reproduction a few months before the natural death of the colony [79]. However, this hypothesis has to be taken cautiously because sexually inactive individuals with embryos in *C*. *inornata* were not significantly larger than the embryogenetic sexually active ones. Further studies on reproductive senescence are needed to clarify this peculiar aspect.

### Annual reproductive cycle

The size frequency distribution of spermaries observed during monthly samples suggests that spermatogenesis of *Caryophyllia inornata* follows an annual cycle, where male germ cells require about 12 months to mature. A similar spermatogenesis has been documented, within the Caryophylliidae family, for the deep coral *Lophelia pertusa* in Norway [62]. On the other hand, oocytes were present in only 5–6 months, showing a shorter oogenesis than *L*. *pertusa* (13–14 months in duration, with one or two months overlapping between cycles) and shorter than the other temperate scleractinians whose reproductive cycle has been studied in detail: *Balanophyllia europaea* [37], *Leptopsammia pruvoti* [40], *Astroides calycularis* [44]. These three species display an oogenesis of about 24 months with an overlap of the gametogenetic cycle. Is not unusual for scleractinian gametogenetic cycle to differ between males and females but the general trend is a much longer oogenesis [11,21,40,80,81] which needs more time and energetic investment with respect to spermatogenesis [37,82].

Our results showed that the annual reproductive cycle of *C*. *inornata* is characterized by oogenetic development and fertilization that take place between February and July and appears to be strongly influenced by seasonal variation in photoperiod and water temperature. The increase of photoperiod and water temperature during the spring and early summer coincides with the maximum development of the gonads and might be a potential cue for sperm release and oocytes fertilization. Variations in seawater temperature are often mentioned as an important phenomenon that controls gametogenetic cycles and planula release in many anthozoans [17,21–25,83]. Fewer studies have been shown that even photoperiod could be involved in the reproduction processes [81,84–86]. Histological techniques do not allow to detect with reasonable accuracy the planulation patterns in *C*. *inornata*. However, the population shows decreased fertility in July, which could indicate the release into the environment of planulae derived from the previous period of fertilization (sexual planulae) and, therefore, a rather short maturation period of planulae. The timing of maturation of sexual planulae is usually of the order of several months, 1–4 months for *L*. *pruvoti* [2] and 4–5 months for *B*. *europaea* [37,38]. In *B*. *elegans*, embryos require 14–15 months of development, presenting an equally long oogenesis [87].

### Size of mature oocytes and fecundity

In order to make a comparison within the genus, it has been considered the maximum oocyte size which was greater in *Caryophyllia inornata* (382 μm) than in *C*. *smithii* (150 μm) [88]. On the other hand, the maximum oocytes size of the deep species *C*. *sequenzae* (450 μm) and *C*. *ambrosia* (700 μm) was greater than *C*. *inornata*, while *C*. *cornuformis* was approximately the same size (350 μm) [10]. Within the genus *Caryophyllia*, the size of mature oocytes could increase with the increase of depth [10]. Large oocytes and subsequent lecitotrophic development are currently recognized as an adaptation to environments such as the oligotrophic abyss [89]. The larval development mode has not yet been determined for *C*. *inornata*, but the small size of oocytes (12–382 μm) could suggests a planktotrophic development of the larvae that generally have a rather long pelagic larval phase and a marked ability to disperse [90].

All the oocytes of *C*. *inornata* were considered potentially fertilizable (therefore mature) as we observed their disappearance after fertilization with oogenesis restricted to a short period of time (February-July). This contrasts with the Mediterranean coral *Leptopsammia pruvoti* whose reproductive cycle has been extensively studied. Fecundity of *L*. *pruvoti* was estimated considering only mature oocytes (size > 340 μm) since two distinct stocks of oocytes are present, resulting in thousands of times lower (20.2 mature oocytes) fecundity than in *C*. *inornata* [40]. These results suggest that our species tends to produce many small oocytes concentrated in a few months a year.

### Fertility

*Caryophyllia inornata* was fertile between 6 and 8 mm in length, the same size of sexual maturity. However, it is noteworthy that in the smaller size (between 4 and 6 mm) almost 50% of polyps was able to produce embryos, while less than 30% of the same size class was sexually mature. It is therefore likely that this species begins to produce embryos before producing germ cells, suggesting again a possible agamic production of brooded embryos. In contrast to the “bell shaped” distribution of sexually mature individuals, the distribution of fertile (embryogenenetic) individuals showed an increasing trend, suggesting that larger/older polyps maintain their ability to produce embryos even without sexual reproduction. Combosch and Vollmer [6] found that bigger colonies of *Pocillopora damicornis* reproduce more asexually than smaller colonies, leading to increased recruitment and survival of the successful genotypes in larval cohorts.

In *C*. *inornata*, 87% of males and 66% of sexually inactive individuals had embryos at different stages of maturation (66% of total individuals). The production of embryos by these individuals was not related with seasonal variations in water temperature and photoperiod. In fact, these embryogenetic polyps showed all stages of embryo development throughout the year [46]. Embryogenetic sexually inactive individuals, that strongly characterize this population, might be: i) a third reproductive state that reproduces only agamically; ii) sexually old individuals (as observed in *Stylophora pistillata*) [79], with the ability to produce agamic embryos; iii) quiescent males during the months immediately following the fertilization period; iv) cryptic females within the group of sexually inactive individuals. In fact, the high proportion of this group raises the possibility that females could be present in the same abundance as the sexually inactive individuals, but that their gametes develop in a shorter period (5–6 month per year). The sea anemones *Actinia equina*, *A*. *tenebrosa* and *A*. *bermudensis* show similarities with *C*. *inornata* as their populations are characterized by embryogenetic females, embryogenetic males, and embryogenetic sexually inactive individuals that brood embryos throughout the year [69,70,74]. It has been hypothesized that these anemones present a rapid sequential hermaphroditism, producing sexual embryos as females, and continue to brood while they switch rapidly (relative to the duration of brooding) into males, passing through an intermediate sexually inactive phase [91]. However, molecular studies and laboratory experiments demonstrate that embryos inside males and sexually inactive individuals may be produced by some form of agamic internal budding [14,15,18,69,72,73,75,92].

The continuous and high fertility of *C*. *inornata* in the study area, on the order of about a hundred embryos per polyp, might partially be due to asexual production of planulae, making this species a successful colonizer. As such, the small oocytes and the consequent planktotrophic development may favor the dispersal and colonization of distant areas. However, the effect of habitat stability and varying levels of disturbance on sexual and asexual reproduction might be more complex [15].

Summarizing, *C*. *inornata* was sexually mature and produced embryos between 6–8 mm in length. Gametogenesis was influenced by temperature and photoperiod and was characterized by a rapid oogenesis. *C*. *inornata* showed small oocytes and high fecundity. In contrast to gametogenesis, fertility did not show a seasonal trend since embryos were found in females, males and sexually inactive individuals throughout the year, suggesting an agamic origin of the embryos. Further analysis with molecular markers such as hypervariable microsatellites are needed to confirm a possible asexual production of brooded embryos in *C*. *inornata* at Elba Isle. Although several studies on the production of brooded embryos have been carried out, the precise nature of this reproductive mode is still unknown.

## Supporting Information

S1 Fig Living specimens of *Caryophyllia inornata* photographed at Elba Isle (42°45’N, 10°24’E). (EPS)

S1 Dataset Full overview of the raw data used for this study. Biometric measurements (length, width, height and volume), reproductive state, oocytes fecundity/spermaries abundance, gonadal index and fertility for each polyp analyzed (N = 158).
(XLSX)

S2 Dataset Environmental data used for this study. Average monthly water temperature (°C) and photoperiod (h) from May 2009 to October 2010 at Elba Isle.
(XLS)

S1 Results Data used to generate Fig 1. Monthly frequency of the 5 reproductive states characterizing the population of Elba Isle. See figure legend in the manuscript.
(XLSX)

S2 Results Data used to generate Figs 2 and 5. Fig 2. Fraction of sexually mature individuals per size class in millimeters. See figure legend in the manuscript. Fig 5. Fraction of fertile individuals per size class in millimeters. See figure legend in the manuscript.
(XLS)

S3 Results Data used to generate Fig 3. Size-frequency distribution of oocytes and of the five stages of spermary maturation in monthly samples. See figure legend in the manuscript.
(XLS)

S4 Results Data used to generate Fig 4. Variation in water temperature and photoperiod, gamete development (monthly mean + SE) and total fertility (monthly mean + SE). See figure legend in the manuscript.
(XLS)

S5 Results Data used to generate Figs 4 and 6. Fig 4. See figure legend in the manuscript. Fig 6. Relationship between water temperature, photoperiod and monthly mean fertility (+SE) of each reproductive state. See figure legend in the manuscript.
(XLSX)
